# Novel human neutralizing mAbs specific for Spike-RBD of SARS-CoV-2

**DOI:** 10.1038/s41598-021-90348-7

**Published:** 2021-05-26

**Authors:** Margherita Passariello, Chiara Gentile, Veronica Ferrucci, Emanuele Sasso, Cinzia Vetrei, Giovanna Fusco, Maurizio Viscardi, Sergio Brandi, Pellegrino Cerino, Nicola Zambrano, Massimo Zollo, Claudia De Lorenzo

**Affiliations:** 1 Ceinge – Biotecnologie Avanzate s.c. a.r.l., Via Gaetano Salvatore 486, 80145 Naples, Italy; 2 Department of Molecular Medicine and Medical Biotechnology, University of Naples “Federico II”, Via Pansini 5, 80131 Napoli, NA Italy; 3 Istituto Zooprofilattico Sperimentale del Mezzogiorno, Via Salute 2, 80055 Portici Naples, Italy

**Keywords:** Biotechnology, Drug discovery, Diseases

## Abstract

Among the therapies against the pandemic SARS-CoV-2 virus, monoclonal Antibodies (mAbs) targeting the Spike glycoprotein represent good candidates to interfere in the Spike/ACE2 interaction, preventing virus cell entry. Since anti-spike mAbs, used individually, might be unable to block the virus entry in the case of resistant mutations, we designed an innovative strategy for the isolation of multiple novel human scFvs specific for the binding domain (RBD) of Spike. By panning a large phage display antibody library on immobilized RBD, we obtained specific binders by eluting with ACE2 in order to identify those scFvs recognizing the epitope of Spike interacting with its receptor. We converted the novel scFvs into full size IgG4, differently from the previously isolated IgG1 mAbs, to avoid unwanted potential side effects of IgG1 potent effector functions on immune system. The novel antibodies specifically bind to RBD in a nanomolar range and interfere in the interaction of Spike with ACE2 receptor, either used as purified protein or when expressed on cells in its native conformation. Furthermore, some of them have neutralizing activity for virus infection in cell cultures by using two different SARS-CoV-2 isolates including the highly contagious VOC 202012/01 variant and could become useful therapeutic tools to fight against the SARS-CoV-2 virus.

## Introduction

The novel coronavirus SARS-CoV-2, responsible for the Severe Acute Respiratory Syndrome known as Covid-19, infects epithelial cells of the respiratory tract, causing typical signs such as fever, dry cough, fatigue and dyspnea^1,2^. A small fraction of patients however progresses towards a severe form of pneumonia requiring the hospitalization or in some cases intensive care unit treatments^3^. The outbreaks of the new pandemic at the end of 2019 have bolstered the research of new vaccines and biomedical treatments urgently needed to prevent or reduce symptoms associated to the new SARS-CoV-2 coronavirus infection^4^.

Coronaviruses are large enveloped viruses, endowed with a positive sense RNA genome, belonging to the family of coronaviridae. Up to date, they were considered pathogens with a veterinary relevance as they were used to infect mammalian and avian species with a limited impact on human beings^5^. Since the beginning of twenty-first century coronaviruses have crossed the barrier between species, leading to Severe Acute Respiratory Syndrome Coronavirus (SARS-CoV) in 2003 and Middle-East Respiratory Syndrome Coronavirus (MERS-CoV) in 2012^6^.

Cell entry of SARS-CoV-2 Coronavirus is mediated by the Spike glycoprotein that is present in multiple copies creating an extensive crown on virus envelope. Spike glycoprotein is a class 1 fusion protein produced as a single polypeptide of about 1300 aminoacids that trimerizes upon folding^7^. It comprises two functional subunits called S1 and S2: S1 represents the apex of the trimer and by its receptor binding domain (RBD), including residues from 331 to 524 of the S protein, promotes the attachment and fusion of virus particles with host membranes; S2 subunit, anchored to the viral membrane, contains a hydrophobic fusion loop and two heptad repeat regions (HR1 and HR2)^8^. The interaction of Spike with the host receptor Angiotensin-Converting Enzyme 2 (ACE2) represents the first step of the infection, followed by proteolysis and conformational changes of Spike translating into the exposure of the fusion loop and its insertion into the target cell membrane with the release of viral genome into the cells^9–11^. Consequently, the RBD of S1 subunit is critical for determining host specificity and cell tropism^12,13^.

To date, many progresses have been done in the scientific approaches against SARS-CoV-2. These include the inhibition of virus entry, fusion or replication, as well as the suppression of excessive inflammatory responses with different drugs such as Chloroquine, Remdesivir and Dexamethasone, respectively^14,15^. Furthermore, three vaccines have been recently approved by FDA for preventative use: BNT162b2 (from Pfizer), AZD1222 (from Astra Zeneca), mRNA-1273 (from Moderna); and several other candidates are being tested by ongoing phase III clinical trials^16–19^.

Among these approaches, monoclonal Antibodies (mAbs) targeting the Spike glycoprotein represent good candidates to interfere in the Spike/ACE2 interaction, preventing virus cell entry^20^. Therefore, the development of anti-Spike mAbs provides a weapon to contain the spreading of the virus, in order to prevent the worsening of the symptoms, especially in high risk patients. Until now, the U.S. Food and Drug Administration (FDA) issued an emergency use authorization (EUA) for the mAb Bamlanivimab (by Eli Lilly and Company's) for the treatment of mild-to-moderate SARS-CoV-2 adult and pediatric patients^21^.

Since anti-spike mAbs, used individually, might be unable to block the virus entry in the case of resistant mutations in the virus, it would be advantageous to generate cocktails of mAbs. To this aim, we designed an innovative strategy for the isolation of novel human anti-Spike-RBD mAbs, by using the phage display technology. The selection was based on two different parallel approaches that allowed for the isolation of a number of antibodies endowed with the ability to bind with high specificity to the RBD domain of Spike protein responsible for the infectivity of the virus^11^ in order to interfere in the binding of Spike RBD to the ACE2 receptor, and to neutralize the virus before its entry into the cells.

We found that some of the novel isolated antibodies specifically bind to RBD domain in a low nanomolar range and they are able to interfere in the interaction of Spike protein with ACE2 receptor, either used as purified protein or when expressed on cells in its native conformation. Furthermore, three of them have shown neutralizing activity for SARS-CoV2 virus infection in cell cultures. These novel candidate mAbs were also used to set up a new detection method to reveal the level of Spike protein in biological samples, useful for diagnosis and monitoring of the viral disease.

## Results

### Selection of human scFvs specific for Spike of SARS-CoV-2

**Figure 1:**
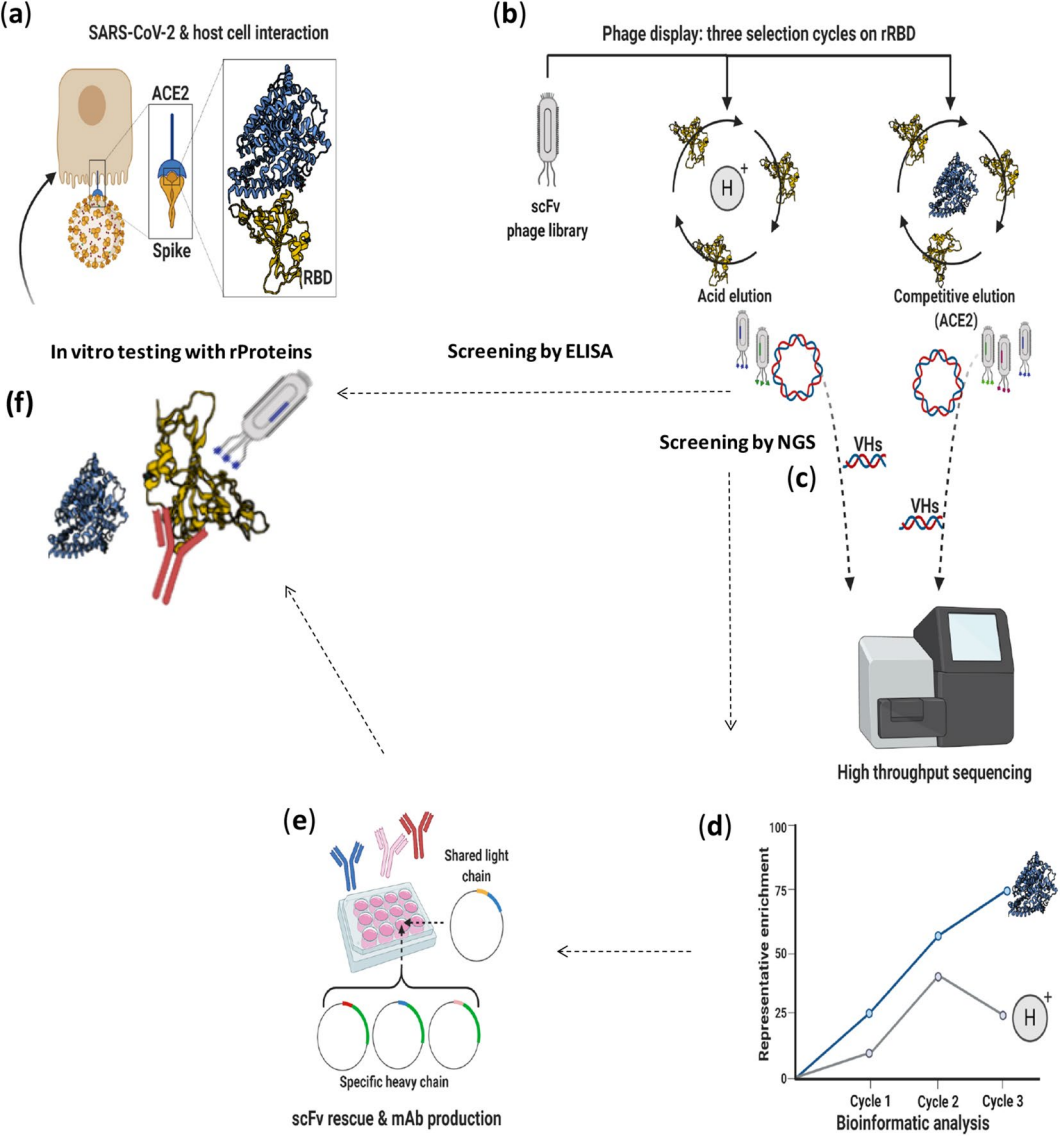
Schematic representation of selection and screening strategies for identification of SARS-CoV-2 neutralizing mAbs. (**a**) Cartoon representing SARS-CoV-2 host cell attachment mediated via ACE2-Spike interaction. The ACE2-RBD interaction in the box is adapted from PDB 6M0J^22^. (**b**) Phage display of scFv library was carried out on recombinant SARS-CoV-2 RBD-Fc protein; acidic or competitive elution were implemented. (**c**) Variable heavy chains of scFvs were extracted from sub-libraries and sequenced by MiSeq Illumina. (**d**) The trend of enrichment of given clones was evaluated within and between the selection cycles. (**e**) Potential binders (scFvs) were converted into fully human IgG4 mAbs in a high yield expressing cell line (HEK293ES_1). (**f**) Binding to rRBD and competition with rACE2 were evaluated in vitro with recombinant protein assay. Neutralization capacity of RBD-specific mAbs was further evaluated for blocking SARS-CoV-2 replication.

The Spike protein is a large transmembrane glycoprotein comprising two subunits: S1 containing a receptor binding domain (RBD), which is responsible for the infectivity of the virus through the interaction with the cell surface ACE2 receptor on human respiratory epithelial cells, and S2 responsible for the membrane fusion in the later step of infection^11,13^ (see Fig. 1a). We designed a novel selection strategy for the isolation of anti-Spike scFv-phages specific for RBD, in order to increase the probability to select mAbs capable of interfering in its interaction with ACE2. To this aim, we performed 4 panning rounds (3 for NGS) on human Spike RBD-Fc recombinant target protein followed by two parallel elution methods: classical acidic elution obtained by lowering the pH and a selective elution, by using the receptor ACE2-Fc chimeric protein which binds to Spike RBD with high affinity to elute those phages that specifically recognize the same epitope (see Fig. 1b). This approach was devised to guarantee an efficient selection of a large number of clones with a high specificity for RBD domain and mapping on the epitope recognized by ACE2, thus more likely endowed with the ability to interfere in the binding of Spike RBD to the ACE2 receptor, and capable of neutralizing the virus before its entry into the cells.

All the selection rounds consisted in two successive negative pannings on the Fc domain, followed by a positive selection on the human chimeric Spike RBD-Fc, to subtract from the collection the phages recognizing the Fc domain present in the chimeric protein. The eluted phages were used to infect *E. Coli* TG1 cells for amplification and further cycles of selection. The strategy of using different elution methods was aimed also at comparing the biological properties of the clones selected by the two strategies, in order to set up a procedure allowing for a novel epitope-specific selection strategy and for obtaining scFvs endowed with specific competitive functional properties.

The screening of positive clones was performed by ELISA (see Fig. 1b,f) and by Next Generation Sequencing (NGS) of the enriched pools of phages from each round of both the acidic and the competitive elution schemes (see Fig. 1c–f).

### Screening by ELISA and expression of positive clones as soluble scFvs

**Figure 2:**
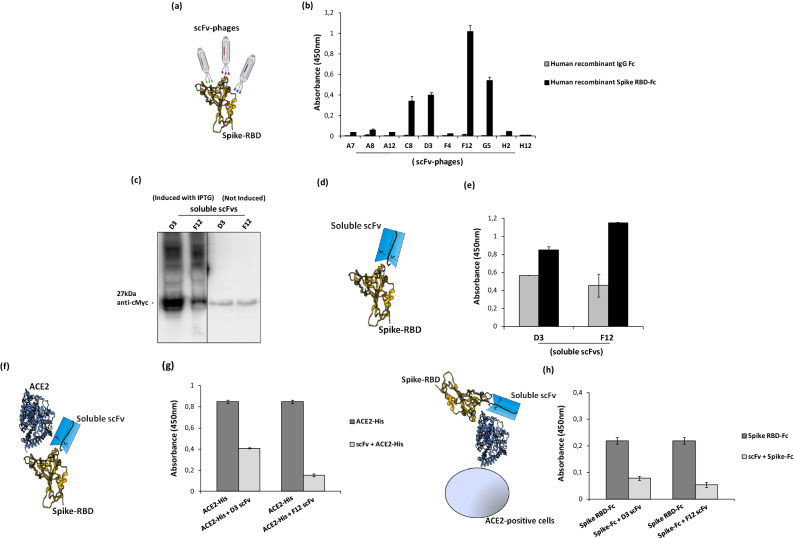
Screening by ELISA and expression of anti-Spike scFvs for analysis of their binding to Spike RBD and their competition with ACE2. (**a**) Representative image of scFv-phages screening by ELISA to test their binding to Spike-RBD recombinant protein. (**b**) Screening of positive phage clones by ELISA assay on human Spike RBD-Fc recombinant protein (black bars) or human recombinant IgG Fc used as a negative control in parallel assays (grey bars). (**c**) Western blotting analysis with the anti-c-myc antibody of the periplasmic extracts of the cells transformed with the two selected positive clones, D3 and F12, expressed in the absence or in the presence of IPTG, used for induction (The blot was obtained by grouping two different parts of the same blot and the black line has been inserted to indicate the two distinct parts. The corresponding full-length blot has been inserted in Supplementary Data set as full-length blot of Figure 2. The samples were processed in parallel in the same experiment). (**d**) Representative image of soluble scFvs binding to Spike-RBD recombinant protein by ELISA (**e**) at two different concentrations: 45 nM (grey bars) or 90 nM (black bars). (**f**) Representative image of soluble scFvs interference in Spike-ACE2 interaction tested by ELISA (**g**). The binding of ACE2-His to immobilized Spike RBD protein in the absence (dark grey bars) or in the presence (light grey bars) of the indicated soluble scFvs (90 nM). (**h**) Competitive ELISA assay was performed by measuring the binding of Spike RBD-Fc protein on ACE2-positive VERO E6 cells in the absence or in the presence of D3 and F12 scFvs.

The scFv phages from the 3rd and 4th rounds of both elution methods were tested by ELISA assays on immobilized Spike RBD protein for the screening of binders. In parallel, ELISA assays were performed also on immobilized human Fc domain to verify the specificity of the binders for RBD (see Fig. 2).

As shown in Fig. 2b, four clones were found capable of specifically binding to RBD of Spike with no significant binding on the Fc, thus confirming the efficiency of the selection strategy. Sequence analyses of those clones revealed that two different scFvs sequences were obtained by the competitive elution, corresponding to the clones, named D3 and F12. To express the positive clones as soluble scFv proteins, the cDNA encoding each scFv was used to transform the bacterial strain SF110 and the expression was induced with Isopropyl β-d-1-thiogalactopyranoside (IPTG). The periplasmic extracts of the two different selected clones were analyzed by Western Blotting^23^ by using the anti-c-myc mAb for detection (Fig. 2c). As shown in Fig. 2c, the scFvs were expressed as soluble proteins of the expected molecular weight of 27 kDa.

### ELISA assays of scFvs on SARS-CoV-2 Spike RBD

To verify whether the soluble scFvs retained the binding properties of the scFvs displayed on phages, they were tested by ELISA assays, on recombinant human Spike RBD-Fc chimeric protein, at increasing concentrations.

The selected anti-Spike soluble scFvs, named D3 and F12, were found able to selectively bind to RBD in a dose dependent manner (Fig. 2e).

To test whether the scFvs could also compete with the ACE2 receptor for the binding to S1 Spike protein, we performed an in vitro competitive ELISA assay^24^ by measuring the binding of ACE2 to immobilized Spike RBD protein in the absence or in the presence of the selected soluble scFvs. As shown in Fig. 2g, the binding of ACE2 in the presence of D3 or F12 used as soluble scFvs was significantly reduced, compared to that observed in their absence, thus suggesting that the novel anti-Spike antibody fragments interfere in the interactions of Spike RBD with ACE2 receptor. To verify whether this ability could be confirmed also on ACE2-positive cells^2^, in vitro competitive ELISA assays were performed on VERO E6 cells^10^, by measuring the binding of Spike RBD-Fc to the cells before or after a preincubation with a five fold molar excess of the selected soluble scFvs, for 2 h at room temperature. As shown in Fig. 2h, the clones D3 and F12 interfere in the interaction of Spike RBD with ACE2 receptor also when it is expressed on the cell surface in its native conformation.

### Identification of enriched scFv sequences by next-generation sequencing and conversion into antibodies

To quickly identify additional monoclonal antibodies targeting Spike protein from SARS-CoV-2, a high-throughput screening was implemented in parallel. The workflow was based on phage display coupled to next generation sequencing and to rapid conversion into fully human antibodies for binding and neutralization screening (Fig. 1). Recombinant RBD-Fc of SARS-CoV-2 was used as bait for panning cycles and to widen the range of possible binders, two different elution methods were exploited in parallel selections. Thus, a non-specific pH-based elution was compared to a competitive one by using ACE2-Fc chimeric protein with the aim to reverse the interaction of those clones with a competitive binding for RBD. To identify potential binders, next generation sequencing was coupled to the screening to find out even those less represented clones within the enriched sub-libraries.

**Figure 3:**
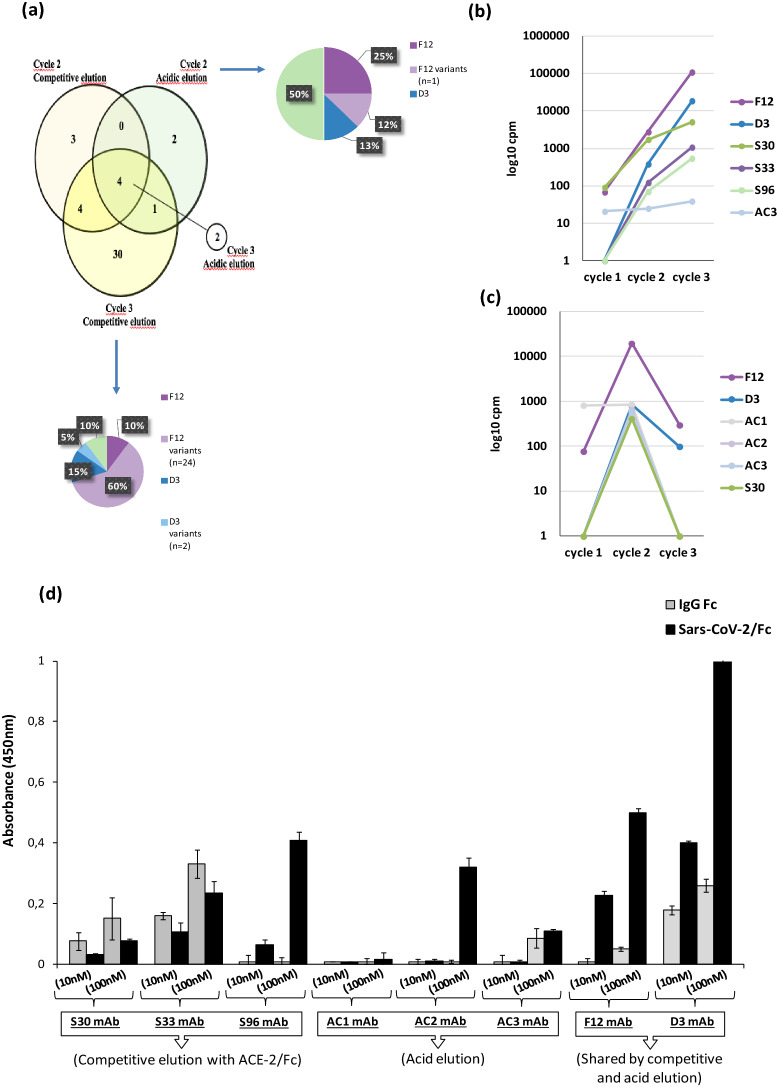
In silico analysis of sub-libraries and identification of potential binders. (**a**) Venn diagram representing the overlapping of full lenght scFvs among top 100 enriched sequences (in-frame and non-Stop codon bearing) between selection cycles from different elution methods (i.e. acidic and competitive). Cycle 3 from acidic elution was represented apart as only two clones resulted enriched. Pie charts represent the distribution of clones within cycle 2 from acidic elution and cycle 3 from competitive one. F12 (dark violet) and D3 (dark blue) resulted as the most enriched ones. Sequences differing from F12 and D3 in only 1 amino acid were indicated as “variants” (light violet and light blue). (**b**,**c**) The enrichment of valid clones between the three selection cycles for competitive elution (**b**) and acidic elution (**c**). (**d**) ELISA assay on human Spike RBD of the converted mAbs: the eight converted mAbs were tested on human Spike RBD-Fc recombinant protein (black bars) or on human Fc (grey bars), used as negative control in parallel assays.

As the adopted phage library of scFvs consists of a few constant scaffold light chains, joined to highly diverse variable heavy chains, we extracted from all the selection cycles the VH region for further analysis of clonal diversity by NGS. Thus, plasmid dsDNA was extracted from phage-infected *E. coli* cells from each round of selection for both elution methods. To discard empty scaffolds or fragmented clones (linker-VL, VH-linker), the 750 bp fragments corresponding to the full length inserts (VH-linker-VL) were isolated by digestion with suitable restriction enzymes. These purified fragments were further digested with *BamHI*, removing almost completely the VL region allowing to run samples in paired end 2 × 300 sequencing in MiSeq Illumina platform. VH fragments from different selection cycles were barcoded in pairs to pull in the same sequencing run cycle_1 & cycle_2 from competitive elution; cycle_1 & cycle_2 from pH elution; cycle_3 from acid & cycle_3 from competitive elution. The reads for each unique fragment were expressed as counts per million (cpm) to compare the trend of enrichment of a given sequence within and between the different selection cycles. Among the top 100 ranking clones, we discarded those that resulted out-of-frame as well as those bearing stop codons. In-frame sequences resulting from these filters, were translated into proteins to merge cmp of clones with silent nucleotide changes. The heterogeneity and the overlapping between selection cycles were analysed. Eleven, 39, 7 and 2 unique in-frame sequences were identified respectively in the cycle 2, cycle 3 from competitive elution and cycle 2 and cycle 3 from acid elution. The cycle 3 from acidic elution led to enrichment of non-informative clones (out-of-frame or stop codon-bearing) and dilution of only two potential binders among them. For this reason, cycle 2 from acid elution and cycle 3 from competitive elution resulted to be the best selection cycles in terms of diversity and numerosity (Fig. 3a). Interestingly, independently from elution method, 4 clones resulted commonly enriched into all the sub-libraries as shown in the Venn diagram in Fig. 3a, including those corresponding to D3 and F12. Moreover, cycle 3 from competitive elution and cycle 2 from acidic one, resulted highly enriched in two shared clones and in a plethora of sequences differing from the latter in only 1 amino acid position.

To improve the chance to identify clones targeting different epitopes of SARS-CoV-2 RBD, the only clones differing each other at least for two amino acids were considered for advanced analysis. Beyond the shared clones, we identified three clones specific for competitive elution, called S30, S33 and S96, and three as derived from acidic elution, hereinafter referred to as AC1, AC2 and AC3.

The trend of enrichment of these clones was analysed for both competitive and acidic elution (Fig. 3b,c). Given the sharing of clones between competitive and acid elution, a total of 8 clones was identified as of interest (D3, F12, S30, S33, S96, AC1, AC2 and AC3) and were rescued by overlapping PCR with HCDR3-specific oligonucleotides from the enriched sub-libraries. The reconstituted scFvs were converted into fully human monoclonal antibodies by sub-cloning the variable domains in frame to human IgG4 Fc into mammalian expression vectors. Recombinant antibodies were produced and purified from HEK293EBNA_SINEUP_1 (abbreviated as HEK293ES_1).

### Characterization of the novel converted anti-spike antibodies

The converted human IgG4 antibodies, generated from the scFvs screened by ELISA assays or identified by NGS as the best ranking Spike protein binders, were tested by ELISA assays on immobilized Spike RBD-Fc or Fc to confirm their binding specificity. As shown in Fig. 3d, F12 and D3 mAbs retained the binding specificity of the parental scFvs, already partially characterized. Among the 6 new identified enriched sequences by NGS, only two, named S96 and AC2, were found able to specifically bind to RBD, whereas the others did not significantly bind to RBD or recognized also the Fc.

**Figure 4:**
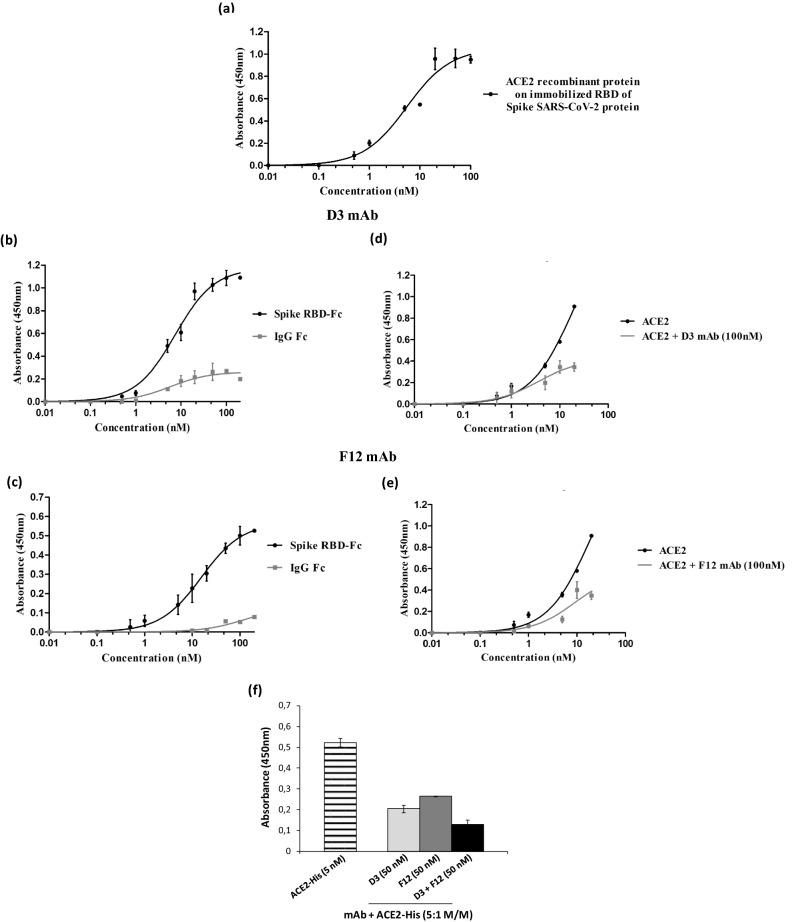
Binding affinity of D3 and F12 mAbs for Spike RBD and their competition with ACE2. (**a**) The binding affinity of ACE2 receptor to immobilized SARS-CoV-2 RBD recombinant protein was tested by ELISA as a positive control. (**b**,**c**) ELISA assays were performed by testing D3 (**b**) and F12 (**c**) mAbs at increasing concentrations (0.5–100 nM) on human Spike RBD-Fc chimeric protein (black curves). In parallel the Fc domain (grey curves) was used as a negative control. (**d**,**e**) Competitive ELISA assays were performed by measuring the binding of ACE2-His protein to Spike RBD in the absence or in the presence of D3 (**d**) and F12 (**e**) mAb used at a concentration of 100 nM. (**f**) Competitive ELISA assays were performed by measuring the binding of ACE2-His to RBD in the absence (striped bar) or in the presence of the indicated mAbs used alone (gray bars) or in combination (black bars) at a concentration of 100 nM. The binding values were reported as the mean of at least three determinations obtained in three independent experiments. Error bars depicted means ± SD*.*

To determine the affinity of these novel mAbs, we firstly determined by ELISA the binding affinity of the recombinant ACE2 protein on immobilized recombinant RBD of Spike SARS-CoV-2 protein (Fig. 4a). The apparent affinity (Kd) measured from the binding curve was found to be 5.5 nM, comparable to that previously reported in literature^22^, thus we then tested D3 and F12 at increasing concentrations by ELISA in the same conditions.

We found that the best binder D3 specifically binds to the human RBD-Fc chimeric protein with high affinity (Kd 7.4 nM), while showing only a poor binding to the Fc domain used as a negative control (Fig. 4b).

To test its ability to interfere in the interaction between Spike RBD and ACE2 receptor, a competitive ELISA assay was also performed on immobilized Spike RBD by measuring the binding of increasing concentrations of ACE2-Fc in the absence or in the presence of 100 nM of D3 mAb. The results, shown in Fig. 4d, indicate that the D3 mAb retains the ability of the parental scFv to interfere in the binding of ACE2 to Spike protein when used at a higher concentration than ACE2.

Similar results were obtained for the novel isolated F12 mAb, tested by ELISA assays on immobilized RBD to analyze its binding affinity and its ability to compete with ACE2 in the interaction with Spike. As reported in Fig. 4c, the antibody binds to the human RBD-Fc chimeric protein with a good affinity (Kd 10.5 nM) and significantly interferes in the binding of Spike to ACE2 receptor (see Fig. 4e).

Considering the inhibitory effects of both these two novel isolated anti-Spike mAbs, we combined them in the competitive ELISA assay, performed as described above. As reported in Fig. 4f, we confirmed that the combination of D3 and F12 antibodies show additive effects in the interference of Spike/ACE2 interaction.

**Figure 5:**
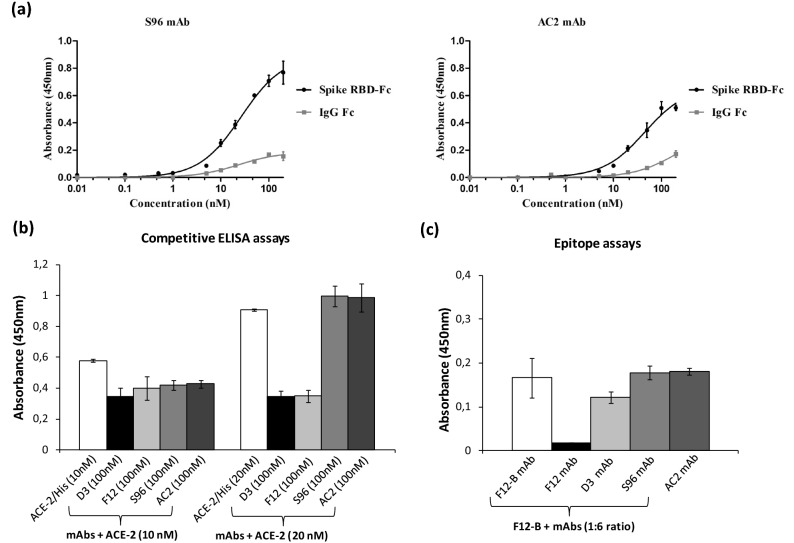
ELISA assays on Spike RBD to test the binding affinity of S96 and AC2 mAbs and their competition with ACE-2. (**a**) The binding affinity and specificity for Spike RBD of S96 and AC2 mAbs was evaluated by testing each mAb at increasing concentrations (0.5–100 nM) on Spike RBD-Fc chimeric protein or Fc, used as a negative control. (**b**) Competitive ELISA assays were performed by measuring the binding of ACE2-His to RBD in the absence (white bars) or in the presence of the indicated mAbs (gray and black bars) at a concentration of 100 nM. (**c**) Competitive ELISA assays to determine the epitope binning were performed by measuring the binding of Biotinylated F12 (F12-B) mAb to RBD, pre-incubated in the absence (white bars) or in the presence of the indicated mAbs (grey and black bars). The binding values were reported as the mean of at least three determinations obtained in three independent experiments. Error bars depicted means ± SD*.*

The two additional antibodies, identified by NGS and named S96 and AC2, were found able to specifically bind to RBD with a lower affinity (Kd of about 26.5 nM and 45 nM for S96 and AC2, respectively) and to be less or not competitive with ACE2 for the binding to RBD with respect to F12 and D3 (see Fig. 5).

To test whether the different mechanisms of action of these two antibody groups is due to the different epitopes recognized, we performed competitive ELISA assays by measuring the binding to immobilized Spike RBD of biotinylated F12 in the absence or in the presence of saturating concentrations of unlabelled F12, D3, S96 or AC2 mAbs. As shown in Fig. 5c, the binding of biotinylated F12 in the presence of S96 or AC2 mAb is not significantly reduced, whereas it is efficiently competed by unlabelled F12, used as a control, thus suggesting that the novel antibodies recognize different epitopes. The D3 mAb partially reduced (of about 25%) the binding of biotinylated F12 to RBD, thus suggesting that these two antibodies likely recognize distinct although overlapping epitopes.

### Cross-reactivity of the novel anti-SARS-CoV-2 mAbs with SARS-CoV

High sequence identity has been reported between the Spike proteins of SARS-CoV-2 and SARS-CoV, in particular in the RBDs where the amino-acid sequence identity reaches 73%^25^. Indeed, patients infected by SARS-CoV-2 were able to produce cross-reactive antibody responses to SARS-CoV RBD^13,26^.

**Figure 6:**
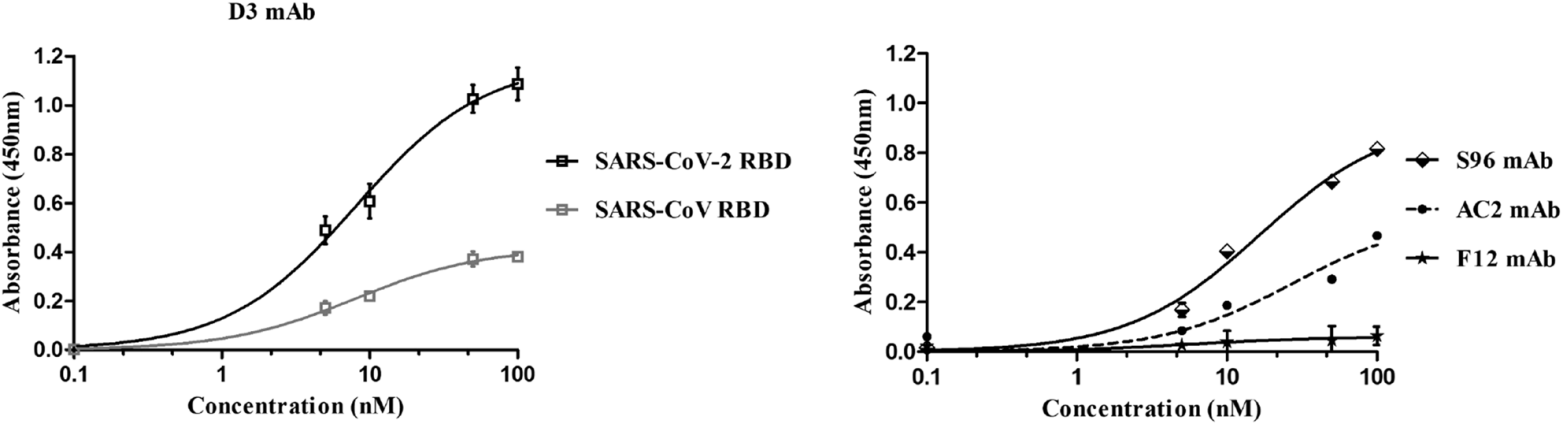
Cross-reactivity of the novel mAbs for SARS-CoV RBD recombinant protein. The binding of D3 (squares, grey curve), S96 (rhomboids, black curve), F12 (stars, black curve) or AC2 (full circles, black curve) mAbs, tested at increasing concentrations (5–100 nM) on immobilized SARS-CoV RBD protein was analyzed in comparison with the binding of D3 to immobilized SARS-CoV-2 RBD protein (empty squares, black curve on the left) by ELISA assays. Both the RBD proteins were coated on multi-well plates at the concentration of 5 μg/ml and treated as described in “Methods” section. The binding values were reported as the mean of at least three determinations obtained in three independent experiments. Error bars depicted means ± SD.

To test whether the novel selected antibodies were cross-reactive for SARS-CoV RBD, we performed ELISA assays on immobilized SARS-CoV RBD by testing each mAb at increasing concentrations. As reported in Fig. 6, the novel anti-Spike F12 mAb does not recognize SARS-CoV RBD whereas the D3 and AC2 mAbs bind to SARS-CoV RBD, even if they show a lower binding ability with respect to that observed on SARS-CoV-2 RBD, tested in parallel assays as a positive control. On the contrary, the binding curve of S96 mAb on SARS-CoV RBD (Fig. 6) is superimposable to that obtained on SARS-CoV-2 RBD (Fig. 4), thus suggesting that this antibody is highly cross-reactive for the other member of the Coronavirus family. Furthermore, these data together with those of competitive ELISA reported above shed light on the epitopes recognized by the novel mAbs. Since F12 and D3 interfere in the binding of RBD to ACE2, they should bind to RBM (corresponding to 438–505 aa sequence of Spike) where the contacts between the two proteins occur. Considering that the antibodies D3 and F12 do not cross-react with the RBD of Spike of SARS-CoV, which shows a high (73%) sequence identity with that of SARS-CoV-2 and differs significantly only in the 455–488 segment (see Supplementary Figure 3), we can conclude that D3 and F12 bind to this specific region, whereas S96 which cross-reacts with the Spike of SARS likely binds to a region with shared sequences outside of this segment.

### SARS-CoV-2 neutralization by the novel human mAbs

The neutralizing activity of D3 was evaluated by preincubating increasing concentrations of the antibody (2–20 μg/ml concentration range) with the virus (0.005–0.5 MOI) for 1 h at 37 °C. Then the mixtures were added to the VERO E6 cells to allow for the infection. After 60–70 h of culture, the cells were harvested, washed and lysed for the extraction of RNA to be analyzed by RT-PCR for measuring the level of N1 viral gene into the cells.

**Figure 7:**
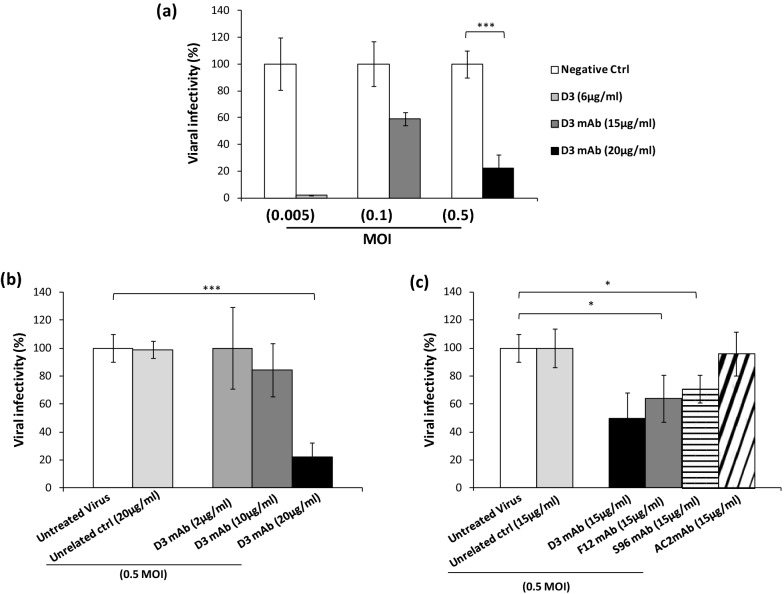
Neutralization assays to test the biological effects of anti-Spike mAbs. VERO E6 cells were infected with the SARS-CoV-2 virus at the indicated MOI, preincubated in the absence or presence of each mAb used at the indicated concentrations. (**a**) Viral cell entry and infectivity was measured by RT-PCR of the cell extracts after washes by analyzing the expression of N1 viral gene. The values are expressed as percentage with respect to the negative untreated control. (**b**) Dose dependent effects of D3 mAb on viral infectivity were measured at the highest viral load. (**c**) Comparison of the efficiency of the different mAbs for inhibiting virus infectivity of cell cultures at a concentration of 15 µg/ml.

As shown in Fig. 7a, the antibody D3 significantly inhibited the cell virus entry by totally blocking it at a concentration of 6 µg/mL when a low viral load was used, and reducing it by 80% at a concentration of 20 µg/mL when a high viral load (0.5 MOI) was used. As shown in Fig. 7b, D3 efficiently inhibits the cell virus entry and consequent infectivity in a dose dependent manner. To compare its effects with those of the other mAbs, we repeated the experiment by testing all of them at a concentration of 15 µg/mL (corresponding to the IC_50_ of D3) with the infecting virus at the highest dose (0.5 MOI). As shown in Fig. 7c, S96 and F12 inhibited the viral infectivity by 30% and 40%, respectively, whereas AC2 did not show significant effects.

**Figure 8:**
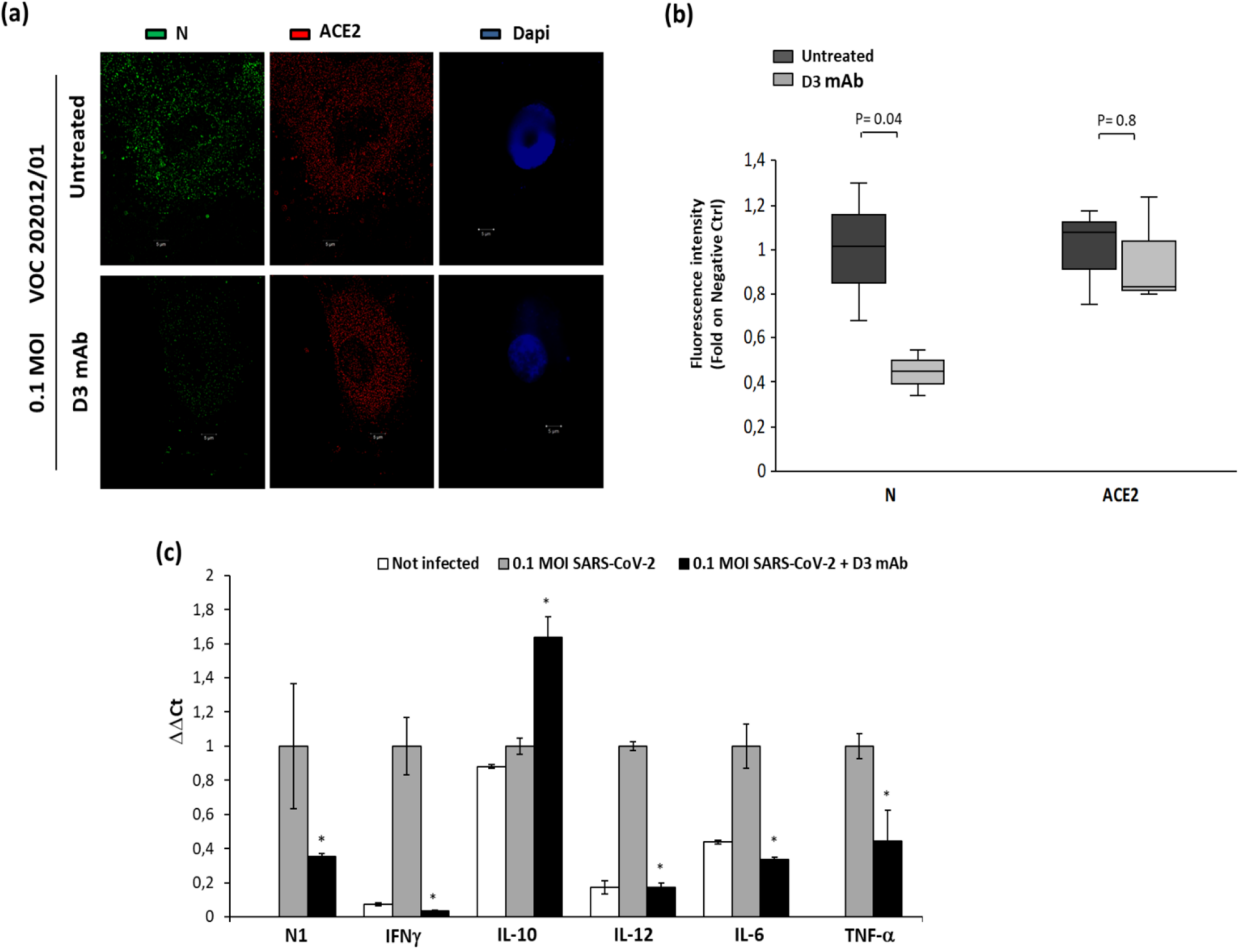
Analysis of human primary cells, infected with the VOC 202012/01 lineage B.1.1.7 variant in the absence or presence of D3, by immunofluorescence assays and RT-PCR. (**a**) The cells untreated or infected with the VOC 202012/01 variant of SARS-CoV-2, in the absence or presence of D3 for 72 h at 37 °C, were fixed, washed and permeabilised, as described in the Methods. After blocking, the slides were incubated with the relevant primary antibodies overnight at 4 °C: anti-ACE2 (1:100; ab15348; Abcam) or anti-SARS-CoV-2 Nucleoprotein (N) Antibody (1:100; No. 35-579, ProSci), followed by the relevant secondary anti-mouse Alexa Fluor 488 (1:200; ab150113; Abcam) and anti-Rabbit Alexa Fluor 546 (1:200; A-11035; ThermoFisher), respectively. DNA was stained with DAPI (1:5000; #62254; Thermo Fisher). Microscopy images were obtained with the Elyra 7 platform (Zeiss) with the optical Lattice SIM technology, using the 63 × oil immersion objective. (**b**) Quantification of the mean fluorescence intensity was performed by using the ZEN software (Zeiss, black edition). (**c**) Cell extracts were analyzed by RT-PCR for measuring the expression of N1 viral gene and the levels of the indicated cytokines. In parallel, the extract of uninfected cells was used as negative control.

The lead neutralizing antibody D3 was further tested for its ability to inhibit cell entry and infectivity of cultures of human primary cells with the UK variant GISAID EPI_ISL_736997 (VOC 202012/01 lineage B.1.1.7) and we found that the antibody retained its neutralizing ability (Fig. 8). In particular, D3 at a concentration of 50 nM significantly reduced the level of Nucleocapsid protein (N) in the infected cells, as shown by immunofluorescence analyses of treated cells. Since this VOC 202012/01 variant contains the mutation D614G, implicated in increased transmissibility of up to 71% and multiple other mutations, including the substitution at position 501 in the RBD region (N501Y) and the following ones outside the RBD: H69del, V70del, Y144del, A570D, P681H, T716I, S982A, D1118H, the D3 antibody could be considered a potential therapeutic tool not only against the SARS-CoV-2 virus but also against its variants.

### Lack of mAb toxicity in cell cultures

To assess potential toxic side effects of the novel anti-RBD mAbs in vitro, we performed cell viability and lysis analyses on VERO E6 cells treated with each neutralizing mAb for 72 h at 37 °C. Cell viability tests were based either on counts by trypan blue or on 3-(4,5-dimethylthiazol-2-yl)-2,5-diphenyltetrazolium bromide test (MTT). Cell lysis was also checked by measuring lactate dehydrogenase (LDH) release in the culture medium. As shown in Supplementary Figure 1, no significant toxic side effects were observed up to the highest concentration of 150 nM (about 25 µg/mL). Furthermore, we quantified multiple cytokines in the extracts of D3-treated and infected human primary cells (Fig. 8) and we found that all the pro-inflammatory cytokines involved in tissue damages, such as IL-6 and TNF were significantly decreased by the treatment with D3, whereas the only cytokine observed at higher levels was IL-10 in line with its anti-inflammatory properties.

### D3 mAb can be used for the detection of Spike protein level in biological samples

To test whether the novel mAbs could be used to develop a new rapid method to detect the level of Spike protein in the saliva biosamples to be used for diagnostic applications, we also set up an ELISA assay. Briefly, immobilized ACE2 was used as a capture molecule and the anti-Spike mAb, was then added to detect the levels of the spike antigen. Thus, the D3 mAb was used by testing known and increasing concentrations of RBD (see Supplementary Figure 2) to obtain a calibration curve to be used for determining the levels of the antigen in the saliva samples. Similar results were obtained also when the RBD or the whole human S1 recombinant protein, used in parallel as a control, were heated at 65 °C for 30 min to mimic the inactivation protocol of the virus in the saliva samples.

## Discussion

SARS-CoV-2 virus caused the Coronavirus disease 2019 (Covid-19) by infecting millions of individuals globally through the recognition of ACE-2 receptor on lung epithelial cells by the viral transmembrane spike protein^27^. Specifically, the Spike protein is made up of two subunits: S1 comprising the RBD domain, mainly involved in the recognition of the receptor target on the host cell surface, and S2 which has a main role in the fusion during the later step of infection^9,12,13^.

In this study, we used an innovative phage display selection strategy to isolate a set of novel human antibodies to fight SARS-CoV-2 viral infection. To this aim we chose as bait for the selection of phage antibodies the recombinant RBD of the Spike protein to increase the probability to obtain neutralizing antibodies capable of interfering in the interaction of Spike with its ACE2 receptor on the cells. As a further innovative step to reach this goal we introduced a competitive elution of the binder phages from RBD by using a large molar excess of ACE2 which has a high affinity (Kd = 5 nM) for Spike in order to guarantee the isolation of phages that specifically recognize the same epitope (RBM) bound by ACE2, as only antibodies recognizing RBM are able to inhibit the interaction of Spike with the receptor and consequent virus cell entry. However, we used in parallel also the conventional acidic elution to collect also all the other potential binders.

The screening of the binders was performed in parallel by ELISA assays and by NGS in order to compare the sequences obtained by the competitive elution strategy with those obtained by the classical method of acidic elution. As a first observation, we obtained a larger number of RBD specific binders from the novel procedure and only one (named AC2) from the conventional method.

Recently, novel human antibodies raised against SARS-CoV-2 spike protein S1 and S2 domains or against SARS-CoV-2 nucleoprotein were obtained and generated as fully human IgG1 antibodies^28–30^. Cross-reactive patterns between SARS-CoV-2 proteins and autoimmune target proteins were found to play a role in the systemic inflammatory response in patients affected by Covid-19 leading to the development of autoimmune diseases post-infection in susceptible subgroups of patients^31^.

The novel scFvs obtained from both our selection strategies and devoid of the Fc, responsible for antibody-dependent enhancement (ADE) possible with human sera from convalescent patients, could be used in this format with no risks of inducing effector functions such as activation of immune cells and complement cascade that could be detrimental in an inflammatory disease, such as Covid-19. However, to increase their stability and avidity we converted them also into full size IgG4, differently from all the previously reported IgG1 mAbs, to avoid unwanted potential harmful side effects of IgG1 endowed with the above mentioned potent effector functions on immune system. Indeed the IgG4 isotype, differently from the IgG1, used for all the previously reported anti-Spike mAbs^20,25,28–30,32^, does not induce sensitization of NK and mast cells and allow to avoid unwanted potential harmful side effects of IgG1, such as the activation of immune cells (NK cells and macrophages) and complement cascade that have been found detrimental in an inflammatory disease, such as Covid-19^33^. Indeed, in the most severe forms of COVID-19, over-activation of the complement system has contributed to the cytokine storm, endothelial inflammation and thrombosis. This aspect represents an absolute and important novelty to obtain safe therapeutic tools with respect to other mAbs developed by companies and many other mAbs reported in literature^28,32,34,35^.

We found that the novel isolated antibodies were able to specifically bind to RBD domain in a nanomolar range and two (named D3 and F12) out of four can interfere in the interaction of Spike protein with ACE2 receptor, either used as purified protein or when expressed on cells in its native conformation. Moreover, by competitive ELISA assays we demonstrated that some of them recognize different epitopes.

When tested for their inhibitory effects on cultures of VERO E6 cells infected with the virus, the antibody D3 displayed the highest neutralization potency, by totally blocking cell virus entry at a concentration of 6 μg/mL when a low viral load was used and by reducing it by 80% at a concentration of 20 μg/mL when a high viral load (0.5 MOI) was used. Interestingly, the only antibody devoid of biological activity against SARS-CoV-2 was found to be AC2, the antibody obtained by the acidic elution of phages, thus suggesting that the novel strategy of competitive elution with ACE2 allowed us to identify not only a larger number of binders but also those endowed with biological effects against the virus, thus confirming the efficiency of our strategy.

The successful use for the first time of this novel competitive elution method demonstrates that it will be possible in the future to select by phage display novel antibodies specific not only for a chosen target but also for a well defined epitope responsible for biological effects. Eventually, the affinity of the antibodies obtained with this novel approach could be then increased by affinity maturation, if higher affinity is required, but their specificity will be assured from the initial step.

Recently many reports in literature have described viral mutations, selected during treatment of infected patients or spreading globally to confer resistance that could render ineffective the treatment with antibodies^34,36,37^. In particular, the VOC 202012/01 variant with the mutation D614G, implicated in increased transmissibility of up to 71% over and above the previous circulating strains of SARS-CoV-2, was derived from the SARS-CoV-2 20B/GR clade (lineage B.1.1.7) and contains multiple mutations, including the substitution at position 501 in the RBD of viral S gene (N501Y) which is the target of the novel mAbs^8,38–40^.

Thus, we further tested D3 for its ability to inhibit the infectivity of cultures of human primary cells with the VOC 202012/01 lineage B.1.1.7 variant and we found that the antibody retained its neutralizing ability with no side toxic or pro-inflammatory effects, thus confirming its potential as a safe therapeutic tool.

Differently from many previous reports based on the use of pseudovirus for cell culture infections^28,35^, we decided to test the novel mAbs directly on two SARS-CoV2 viral isolates including the highly contagious variant (VOC 202012/01) for testing their neutralizing potency in the real life therapeutic-like context, but this makes it difficult to compare their anti-viral efficacy with that of previously reported mAbs tested in different models, even though they have shown high affinity for Spike^25,28–30,32^.

In order to test whether the novel antibodies could be used also for inhibiting other members of Coronavirus family, we tested them for their binding to Spike-RBD of SARS-CoV virus and we found that one of them (S96) showed significant crossreactivity. Furthermore, we used the D3 mAb to set up an ELISA detection method to reveal the level of RBD and Spike protein in biological samples that could be useful for the viral detection in saliva samples.

Finally, since we generated a set of fully human anti-SARS-CoV-2 scFvs and neutralizing full length antibodies targeting distinct epitopes on the spike RBD, they can be also combined to improve treatment efficacy and to reduce the risk of treatment-escape of viral variants^34^.

## Methods

### Bacterial strains, culture media and antibiotics

*E.Coli TG1* bacterial strain was used for the production of phages; *E.Coli SF110* bacterial strain was used for the expression of the scFvs as soluble proteins. Bacterial growth was carried out in the culture media YT-Medium (A0981) and LB (A0954) from AppliChen Darmstadt, Germany. The Antibiotics used were Ampicillin (A7492) at the concentration of 100 μg/ml and Kanamycin (A1493) at the concentration of 25 μg/ml (from AppliChen Darmstadt Germany).

### Eukaryotic cell cultures

The VERO E6 cells (ATCC #C1008) were cultured in Dulbecco’s Modified Eagle’s Medium (GibcoTM DMEM, Thermo Fisher Scientific 11965 Ferentino Italy), supplemented with 10% (vol/vol) heat-inactivated fetal bovine serum (FBS, Sigma-Aldrich F2442, USA), 50 UI mL^-1^ Penicillin Streptomycin (Gibco Life Technologies 15140-122, Grand Island USA) and 2 nM L-glutamine (Gibco Life Technologies 25030-024, Paisley UK). Freshly isolated human nasal epithelial cells were collected by nasal brushing of healthy donors. The ACE2 gene locus of the nasal epithelial cells underwent next-generation sequencing and was shown not to have any modifier variants (synonymous_variant:c.2247G > A,p.Val749Val;intron_variant:c.1297 + 68_1297 + 69insCTT;splice_region_variant&intron_variant:c.439 + 4G > A; whole exome sequencing data deposited with the European Variation Archive (EVA)—EMBL-EBI; Project ID: PRJEB42411; Analyses: ERZ1700617). These cells were cultured in PneumaCult (#05009; StemCell Technologies USA) with 2 mM L-glutamine (Gibco Life Technologies 25030-024, Paisley UK) and 1% penicillin/streptomycin (P0781; Sigma USA). Cell cultures were grown at 37 °C as previously described^41^.

### Antibodies and human recombinant proteins

The following human recombinant proteins were used: SARS-CoV-2 (2019-nCoV) chimeric Spike RBD-Fc protein, SARS-CoV-2 (2019-nCoV) Spike RBD-His protein and ACE2 His Protein (all from Sino Biological, 10108-H08H Düsseldorfer Eschborn, Germany); Recombinant human coronavirus SARS-CoV-2 Spike Glycoprotein S1 (from Abcam, ab273068, Cambridge UK); Recombinant ACE-2 Fc chimeric protein (Z03484 GenScript, Piscataway NJ); recombinant IgG1 Fc protein (R & D Systems, 110-HG Minneapolis MN).

The following antibodies were used: HRP conjugated anti-His antibody (Qiagen, 1014992 Hilden, Germany); HRP conjugated anti-cmyc antibody (Milteny Biotec, 130-092-113 GmbH Friedrich-Ebert-Strabe Germany); HRP conjugated anti-human Fc antibody (Sigma, AP113P USA); anti-human IgG (Fab’)2 goat monoclonal antibody (Abcam, Cambridge UK ab98535).

### Selection of scFv-phage clones

Phagemid particles were isolated from the library by using the M13-K07 helper phage (Invitrogen, Thermo Fisher Scientific Carlsbad, CA 92008, USA), as previously reported^42^. To isolate anti-Spike scFv-phages, a phagemid library of up 10^12^ different clones was used for selection on human chimeric Spike RBD-Fc protein^43^. For each round of selection, phages were firstly blocked with 5% (wt/vol) Skim Milk Powder in PBS (Fluka Analytical, Sigma-Aldrich, A08300500 USA) and then two negative pannings were performed by incubating them on immobilized rhIgG1-Fc protein domain in 2.5% (wt/vol) Skim Milk Powder for 2 h at 4 °C by gently rotation. The unbound phages were collected in the supernatant by centrifugation at 1200 rpm for 10 min and then incubated with immobilized Spike RBD-Fc protein (20 μg/ml) in 2.5% (wt/vol) Skim Milk Powder over night at 4 °C by gently rotation for the positive selection. After extensive washes with PBS, the bound phages were eluted from the Spike RBD-Fc protein by using two parallel elution methods: the classical elution with 76 mM citric acid (pH 2.5) in PBS for 5 min followed by the addition of 1 M Tris-HCl (pH 8.0) neutralization buffer^23,43^, or selective competitive elution, by using the chimeric receptor ACE2-Fc (100 μg/ml in PBS) which binds to Spike-RBD with high affinity to obtain those phages that specifically recognize the same epitope. The eluted phages were used to infect *E. coli* TG1 cells for amplification and further rounds of selection on the purified chimeric protein. Finally, the collected phages were stored at 4 °C until use.

### Screening of positive clones by ELISA assays

To prepare phages for screening of positive clones by ELISA assay, TG1 cells were infected with the phages eluted from the 3rd and 4th rounds of selection and plated on 2xTY/agar containing glucose (1%) and ampicillin (100 μg/ml) to obtain single separated bacterial clones. The clones were picked and grown into 96-well plates in 2×TY medium containing 1% glucose and ampicillin (100 μg/ml) for 18 h in agitation at 37 °C. A superinfection with M13-K07 helper phage was performed to produce the scFv-phages, that were used for ELISA assays on immobilized Spike RBD-Fc chimeric protein, as previously reported^23,42,44^.

### DNA fragment preparation and libraries generation for high-throughput sequencing

For each cycle, the scFv-containing double strand DNAs were purified from superinfected *E. coli* TG1 cell cultures using Endo free Plasmid Maxi Kit (Qiagen, 12362, Hilden Germany). Heavy chain fragments were isolated by two-step restriction process, as previously described^24^. Briefly, full-length scFv_s_ (VH-Linker-VL) were extracted by using *Nco*I (R3193L New England Biolabs, Ipswich, Massachusetts, USA) and *Not*I (R3189L New England Biolabs, Massachusetts USA) and purified from 1.5% agarose gel using Wizard SV Gel and PCR Clean-Up System (Promega, A9282, Wisconsin USA). To generate fragments suitable for Next Generation Sequencing (NGS), a portion of variable light (VL) chain was removed from full-length scFv_s_ by digestion with *Bam*HI (R3136S New England Biolabs, Massachusetts USA) and the obtained fragment was purified from 1.5% agarose gel using Wizard SV Gel and PCR Clean-Up System (Promega, REF A9282, Wisconsin USA).

Library preparation for sequencing and preliminary bioinformatic analysis of the data were performed at the Center for Translational Genomics and Bioinformatics, Hospital San Raffaele, Milano, Italy, as previously reported^45,46^.

Briefly, isolated VH fragments from each cycle were bar-coded by TruSeq ChIP sample prep kit (Illumina, 15023092) and sequenced to a final concentration of 10 pM with 2 × 300 nt SBS kit v3 on an Illumina MiSeq apparatus. For each sub-library, raw counts were normalized to the total number of counts, obtaining count per million (cpm) values. Obtained nucleotide sequences were translated, taking into account the correct open reading frame.

### scFv reconstitution, antibodies production and purification

The clones of interest were isolated from competitive elution cycle_3 and acidic elution cycle_2 by overlapping PCR, as previously described^43^ and reported in the Supplementary Information.

### ELISA assays

To confirm the binding specificity of the novel anti-Spike soluble scFvs or purified antibodies, ELISA assays were performed on the chimeric Spike RBD-Fc protein. The NuncTM flat bottom 96-well plates (Thermo Fisher Scientific 439454 Ferentino Italy) were coated with 5 μg/mL Spike RBD-Fc recombinant protein in a solution of 0.05 M NaHCO3 for 72 h at 4 °C. After blocking of the coated 96-well plates with 5% nonfat dry milk in PBS for 1 h at 37 °C, the D3 and F12 soluble scFvs or purified mAbs were added at increasing concentrations to the plates in 3% Bovine Serum Albumin (BSA Sigma A7030, St Louise MO) in PBS and incubated for 2 h at RT by gently shaking. After the first incubation, extensive washes were carried out with PBS, and the plates were incubated for 1 h at RT with anti-cmyc or anti-Fab HRP-conjugated antibody, for the detection of soluble scFvs or antibodies, respectively. After washes the plates were incubated with 3,3’,5,5’-tetramethylbenzidine (TMB Sigma-Aldrich T0440, St Louise MO) reagent, as previously reported^24,46–49^. Absorbance at 450 nm was measured by the Envision plate reader (Perkin Elmer, 2102). Binding values were reported as the mean of at least three determinations obtained in three independent experiments.

The Kd values were calculated by the analysis of binding curves of the selected antibodies with the Graphpad Prism software, as previously reported^23^. The equation model used was: Y = Bmax*X/(Kd + X) + NS*X + Background. Bmax is the maximum specific binding in the same units as Y; Kd is the equilibrium binding constant, in the same units as X, and it is the ligand concentration needed to achieve a half-maximum binding at equilibrium; NS is the slope of non-specific binding in Y units divided by X units; background is the amount of nonspecific binding with no added ligand^24^.

### Competitive ELISA assays

In order to investigate the ability of the novel isolated D3 and F12 scFv clones to interfere in the binding of Spike protein to ACE2 receptor, competitive ELISA assays were performed on immobilized Spike RBD-Fc protein or on ACE2-positive VERO E6 cells. To this aim, the ELISA assays on chimeric protein were performed by coating 5 µg/ml of Spike RBD-Fc recombinant protein as described above in 0.005 M NaHCO_3_ solution in a NuncTM flat-bottom 96-well plate for 72 h at 4 °C.

D3 or F12 soluble scFvs, preincubated (fivefold molar excess) with ACE2-His recombinant protein for 2 h at RT, were added to the coated plate for 2 h in agitation at RT. The plates were washed with PBS and the binding of ACE2 to immobilized Spike protein, in the absence or in the presence of the selected soluble scFvs, was measured by incubating the plate with the HRP conjugated anti-His antibody for 1 h in agitation at RT. After washes TMB reagent was added and the signals analyzed as described above. To test the ability of the selected clones to interfere in the binding of Spike protein to ACE2 receptor expressed on VERO E6 cells, cell ELISA assays were performed by plating the cells in round-bottom 96-well plates (2 × 10^5^ cells or for each well). The D3 or F12 soluble scFvs were preincubated with Spike-Fc protein (at fivefold molar excess) for 2 h at RT and then added to the cells for 2 h in agitation at RT. After washes the binding of Spike to the cells, in the absence or in the presence of the selected soluble scFvs, was measured by incubating the plate with the HRP conjugated anti-IgG Fc antibody, and analyzed as described above.

To confirm the ability of the converted IgG4 antibodies to interfere in the interaction between Spike RBD and ACE2 receptor, the competitive ELISA assays were also performed on immobilized Spike RBD-Fc recombinant protein by measuring the binding of increasing concentrations of ACE2-His, in the absence or in the presence of each mAb. After blocking, the coated plates were preincubated with the mAbs used at the concentration of 100 nM for 2 h in agitation at RT. Then, increasing concentrations of ACE2-His recombinant protein were incubated for 2 h ours in agitation. The plates were washed and the binding of ACE2-His was measured by incubating the plate with the HRP-conjugated anti-His antibody for 1 h in agitation at RT, and analyzed as described above.

To evaluate whether the epitopes recognized by the novel isolated anti-Spike mAbs are different, competitive ELISA assays were performed on immobilized Spike RBD-Fc protein (5 μg/mL) by measuring the binding of the biotinylated F12 mAb (Linghtning-Link Biotin conjugation Kit Type B, Innova Biosciences 715-0010, Cambridge UK) to the protein. The biotinylated F12 mAb was tested at increasing concentrations, in the absence or in the presence of saturating concentrations of unlabelled D3, S96 or AC2 mAbs (300 nM) preincubated over night at 4 °C. After washes, the binding of the biotinylated F12 mAb was detected with HRP-conjugated Streptavidin (Biorad 710005, Kidlington UK) and the absorbance measured as reported above.

### In-vitro infection

SARS-CoV-2 viral particles were obtained from a frozen swab from two patients positive for COVID-19 (**Italian Variant**, Genbank MT682732.1, GISAID EPI_ISL_477204; **UK variant**, VOC 202012/01 lineage B.1.1.7, GISAID EPI_ISL_736997). SARS-CoV-2 particles at different multiplicity of infection (i.e. 0.05–0.5 MOI) were preincubated with the antibodies (D3, F12, S96, AC2) at 37 °C for 1 h and then added to Vero E6 (8 × 10^5^/flask) or human primary nasal epithelial cells (3 × 10^5^/flask). Non-infected cells were used as the negative infection control. After 72 h of infection, the cells were lysed and their RNA was extracted and analyzed by Real-time RT-PCR assays, as described in the Supplementary Information. These experiments were performed in a BLS3 authorised laboratory.

*The Spike in VOC 202012/01 variant contains the following mutations:* H69del, V70del, Y144del, N501Y, A570D, D614G, P681H, T716I, S982A, D1118H.

### Immunofluorescence

After the treatment for 72 h with D3 and *VOC 202012/01*, the cells (1 × 10^4^) were fixed in 4% paraformaldehyde in PBS for 30 min, washed three times with PBS, and permeabilised with 0.1% Triton X-100 (215680010; Acros Organics) diluted in PBS, for 15 min. The cells were then washed with PBS and blocked with 3% bovine serum albumin (A9418; Sigma) in PBS for 1 h at room temperature. The samples were incubated for 3 h at room temperature with the relevant primary antibodies: anti-ACE2 (1:100; ab15348; Abcam) or anti-SARS-CoV-2 Nucleoprotein (N) Antibody [3851] (1:100; No. 35-579, ProSci). After washing twice with PBS, the samples were incubated at room temperature for 1 h with the relevant secondary antibody: anti-mouse Alexa Fluor 488 (1:200; ab150113; Abcam) and anti-Rabbit Alexa Fluor 546 (1:200; A-11035; ThermoFisher), respectively. DNA was stained with DAPI (1:5000; #62254; Thermo Fisher) for 10 min at room temperature. The slides were washed and mounted with cover slips using 50% glycerol (G5150; Sigma-Aldrich). Microscopy images were obtained by the Elyra 7 platform (Zeiss) with the optical Lattice SIM technology, using the 63 × oil immersion objective. The quantification of the fluorescence intensity was performed by using the ZEN software (Zeiss, black edition).

### Statistical analyses

Error bars were calculated on the basis of the results obtained by at least three independent experiments and represent means ± SD. For these studies, differences between the experiments were assessed by two-tailed Student’s t-test and the statistical significance was defined as ***p ≤ 0.001; **p < 0.01; *p < 0.05. A probability (*p*) < 0.05 was considered statistically significant.

### Ethics declarations

The only human samples (primary nasal cells), used in this study, were obtained from healthy donors with their approval and informed consent (Protocol n. 157/20, date 22/04/2020 GENECOVID) and used only in BLS3 authorized laboratory. The experimental procedures relative to the use of SARS-CoV-2 in BLS3 laboratory were authorized by Ministry of Health (Ministero della Salute) and by Department of Molecular Medicine and Medical Biotechnology, University of Naples Federico II and by Azienda Ospedaliera Universitaria Federico II, Direzione Sanitaria (protocol n. 0007133 of 08/05/2020). All methods were carried out in accordance with relevant guidelines and regulations of University of Naples “Federico II”.

## Supplementary Information

Supplementary Information.

## Data Availability

Gene sequences accession numbers: GISAID: https://www.epicov.org/. Italian Variant, GISAID EPI_ISL_477204. UK variant, VOC 202012/01, GISAID EPI_ISL_736997. *Genebank* Italian Variant: MT682732.1 (Severe acute respiratory syndrome coronavirus 2 isolate SARS-CoV-2/human/ITA/Naples/2020, complete genome). *Whole exome sequencing data* Whole exome sequencing data of primary human nasal-brushing derived cells were deposited to European Variation Archive (EVA)—EMBL-EBI; Project ID: PRJEB42411; Analyses: ERZ1700617.
