# Supplementary material for: Novel human neutralizing mAbs specific for Spike-RBD of SARS-CoV-2
Source: Sci Rep. 2021 May 26;11:11046. doi: 10.1038/s41598-021-90348-7 (PMC8155001; doi:10.1038/s41598-021-90348-7)
Supplement: Supplementary file 1 — Supplementary Information. [file 41598_2021_90348_MOESM1_ESM.docx]

**TITLE: “Novel human neutralizing mAbs specific for Spike-RBD of SARS-CoV-2”**

**AUTHORS:** ^±^Margherita Passariello^1^, ^±^Chiara Gentile^2^, Veronica Ferrucci^2^, Emanuele Sasso^1^, Cinzia Vetrei^2^, Giovanna Fusco^3^, Maurizio Viscardi^3^, Sergio Brandi^3^, Pellegrino Cerino^3^, Nicola Zambrano^1,2^*, Massimo Zollo^1,2^* and Claudia De Lorenzo^1,2^*

**Affiliations:**

1. Ceinge – Biotecnologie Avanzate s.c. a.r.l., via Gaetano Salvatore 486, 80145, Naples, Italy.

2. Department of Molecular Medicine and Medical Biotechnology, University of Naples “Federico II”, Via Pansini 5, 80131 Napoli (NA), Italy.

3. ‎Istituto Zooprofilattico Sperimentale del Mezzogiorno, via Salute 2, 80055 Portici Naples, Italia

*Correspondence to: Claudia De Lorenzo (cladelor@unina.it; phone: +39-081 3737868), Massimo Zollo (massimo.zollo@unina.it) and Nicola Zambrano (zambrano@unina.it). Department of Molecular Medicine and Medical Biotechnology, University of Naples “Federico II, Via Pansini 5, 80131 Napoli (NA), Italy.

^±^These authors contributed equally to this work.

**Supplementary Figures**

**Supplementary Figure 1.** Cytotoxic effects on VERO E6 cells of the anti-SARS-CoV2 mAbs. (**a**) Percentage of cell viability after 72 h incubation with each indicated anti-Spike mAb (grey bars) compared to those of control untreated cells or cells treated with an unrelated IgG4 (black bars). (**b**) Percentage cell lysis after incubation with each indicated anti-Spike mAb was measured by assessing LDH release; as positive control, cells were treated with 1 % Triton X-100 (empty bar). Error bars indicate SD.

**Supplementary Figure 2.** Detection of the levels of Spike protein in biological samples by using D3 mAb. Immobilized ACE2 was used as capture molecule for Spike-RBD and the anti-Spike D3 mAb, was then added to detect the levels of the spike antigen. D3 mAb was used by testing known and increasing concentrations of RBD to obtain a calibration curve to be used for determining the levels of the antigen in the biological samples.

**Supplementary Figure 3.** Alignment of the aa sequence of SARS-CoV-2 RBD with that of SARS-CoV. The Spike-RBD region of SARS-CoV-2, involved in ACE2 binding (from 319 to 541), shows high homology (evidenced in light gray) with that of SARS-CoV; the RBM domains (corresponding to those included in the black box) contain different residues (marked in dark grey). In brackets is evidenced the epitope recognized by D3 and F12.

**Supplementary Dataset**

Full-length blot of **Figure 2**

Since the blot of **Figure 2c** was obtained by grouping two different parts of the same blot and the black line has been inserted to indicate the two distinct parts, the full-length blot has been inserted here. The samples were processed in parallel in the same experiment

**Supplementary Methods**

**Analysis of positive clones and expression of soluble scFvs**

The positive clones selected by ELISA assays were grown in 2xTY medium containing glucose (1%) and Ampicillin (100 μg/ml) to extract the plasmidic DNA by using QIAprep Spin Miniprep Kit (QIAGEN, 27106 Venlo Netherlands). To determine the number of positive clones containing the cDNA encoding the full lenght scFv, the selected plasmidic DNA was digested from pHEN2 vector with *NcoI* and *NotI* restriction enzymes (R3193L and R3189L, respectively from New England Biolabs, Massachusetts USA) and analyzed by electrophoresis on 1% agarose gel. The positive clones showing the full length insert were prepared for sequencing by using the LMB3 forward and the fdSeq1 reverse primers, respectively complementary to the sequence located upstream the heavy variable (VH) region sequence and downstream to the c-myc-tag sequence.

To express the selected positive clones as soluble scFv molecules, *E. Coli* SF110 cells were infected with each of the anti-Spike scFv-phage preparations and grown in 2xTY medium containing ampicillin (100 μg/ml). To express soluble scFv, the Isopropyl β-D-1-thiogalactopyranoside (IPTG AppliChen A4773, Darmstadt Germany) was added at the concentration of 1 mM (16 hours at R T) when the optical density of the cell culture reached the absorbency of 0.8. Cells were harvested by centrifugation at 3000 rpm for 20 minutes and resuspended in *B-PER* (ThermoFisher 78248 Meridian Rockford USA) for 20 minutes by gently rotation at room temperature (RT). Cell suspensions were then centrifuged at 12000 rpm for 30 minutes at 4°C to obtain the periplasmic extract. Soluble scFvs were analyzed by Western Blotting detected by using a mouse HRP-conjugated anti-c-myc-tag monoclonal antibody.

**scFv reconstitution, antibodies production and purification**

The clones of interest were isolated from competitive elution cycle_3 and acidic elution cycle_2 by overlapping PCR, as previously described^45^. Briefly, to obtain separate VH and VL fragments, two different PCR reactions were performed by using Phusion High-Fidelity DNA Polymerase (Thermo Fisher Scientific, F530S, Massachusetts USA) and clone-specific primers designed within each HCDR3 and in the constant region of plasmid upstream and downstream of VH and VL. To obtain full-length scFv_s_, VH and VL fragments were mixed and extended by HCDR3 overlapping PCR. Recovered scFv_s_ were converted into whole human IgG4 antibodies by cloning the corresponding VH and VL cDNAs in the SINEUP-competent 8.2VH and 4.2VL pEU vectors, expressing the constant heavy and light chains respectively^47^. Briefly, VHs and VLs were amplified by CloneAmp HiFi PCR Premix using specific oligos. In-Fusion HD cloning kit (Clontech Laboratories, 639692, California USA) was used to clone VH in *Bam*HI (R3136S) and *Bss*HII (R0119L) linearized pEU8.2VH vector, and VL in *Apa*LI (R0507L) and *Avr*II (R0174) linearized pEU4.2VL vector. All restriction enzymes were from New England Biolabs, Massachusetts USA.

The obtained vectors were transformed into Stellar Competent Cells (Clontech Laboratories, 636763, California USA) and the resulting colonies were screened by sequence analysis. The vectors containing the correct DNA sequences were prepared with an endotoxin-free system (EndoFree Plasmid Maxi Kit, Qiagen, 12362).

Antibodies were produced co-transfecting VH and VL expressing vectors by using Lipofectamine Transfection Reagent (LifeTechnologies, Inc. 11668019, California USA) into the production enhanced cell line HEK293EBNA SINEUP (HEK293_ES1), expressing the long non-coding SINEUP targeting heavy and light chain signal peptide on mRNAs^48^. Transfected cells were grown for 10 days at 37 °C in CHO medium (Gibco, LifeTechnologes, Inc. 10743029, Waltham, Massachusetts, USA) supplemented with 1% L-glutamine 200 mM (Gibco, LifeTechnologies, A2916801, Massachusetts USA) and 1% Penicillin-Streptomycin 10,000 U/mL (Sigma-Aldrich, P0781, Missouri USA). The antibodies were purified from the conditioned media by using Protein A HP SpinTrap30 (GE Healthcare Life Science, 28-9031-32, Chicago USA), following manufacturer’s instruction. Desalting and buffer-exchange were performed by using PD-10 Column (GE Healthcare Life Sciences, 17085101, Chicago, Illinois, USA). Antibodies were sterilized by filtration with 0.22 μm durapore hydrophilic filters (Millipore, SLGS033SS, Massachusetts USA) and stored in aliquots at -80 °C.

**Real-time RT-PCR assays**

*N1 detection*

RNA samples were extracted with TRIzol RNA Isolation Reagent (#15596018; Ambion, Thermo Fisher Scientific), according to the manufacturer instructions. Real-time RT-PCR was performed using ‘quanty COVID-19’ kits (Ref. RT-25; Clonit; US Food and Drug Administration ‘*in-vitro* diagnostic’ (IVD) approved). These kits allow specific quantitative detection of N1 fragment (from the SARS-CoV-2 N gene), using differentially labelled target probes. These runs were performed on a PCR machine (CFX96; BioRad) under the following conditions: 25 °C for 2 min; 50 °C for 15 min; 95 °C for 2 min; 95 °C for 3 s; 55°C for 30 s (×45 cycles). Cq values of N1 were calculated as means ±standard deviation, as the ratios to the internal control detected with the CLONIT quanty COVID-19 kits.

*N1 and cytokines detection*

RNA samples were extracted with TRIzol RNA Isolation Reagent (#15596018; Ambion, Thermo Fisher Scientific), according to the manufacturer instructions. Reverse transcription was carried out by using ‘5× All-in-one RT Mastermix’ (#g486; ABM), following the manufacturer instructions. The cDNA preparation was analyzed through the cycling method, as follows: incubation of the complete reaction mix at 25 °C for 5 min; at 42 °C for 30 min; at 85 °C for 5 min; and hold at 4 °C. The reverse transcription products (cDNA) were amplified by quantitative real-time PCR using a real-time PCR system (7900; Applied Biosystems, Foster City, CA, USA). The relative expression of the target genes was determined using the 2−ΔΔCt method as fold over vehicle-SARS-CoV-2 infected control. The data are presented as 2−ΔΔCt means ±standard deviations of two to three replicates. The target genes were detected using a Brightgreen 2× qPCR Mastermix low-rox (#Mastermix-lr; ABM.). The details of the primers used in these assays are listed:

| **Gene** | **Primer** | **Primer sequence** |
| --- | --- | --- |
| N1* | Forward | GACCCCAAAATCAGCGAAAT |
|  | Reverse | TCTGGTTACTGCCAGTTGAATCTG |
| IL-6 | Forward | GCCACTCACCTCTTCAGAAC |
|  | Reverse | AGCATCCATCTTTTTCAGCC |
| IL-10 | Forward | CCTGCCTAACATGCTTCGAGA |
|  | Reverse | TGTCCAGCTGATCCTTCATTTG |
| IL-12 | Forward | TGATGGCCCTGTGCCTTAGT |
|  | Reverse | GGATCCATCAGAAGCTTTGCA |
| IFN-γ | Forward | AGGCATTTTGAAGAATTGGAAAGA |
|  | Reverse | AGTAAAAGGAGACAATTTGGCTCT |
| TNF-α | Forward | TCTCTCTAATCAGCCCTCTGG |
|  | Reverse | GCTACATGGGCTACAGGC |

* approved by CDC

**In vitro cytotoxicity assays**

To test the cytotoxic effects of the novel generated IgG4 anti-Spike mAbs, VERO E6 cells were plated in 96-well flat bottom plates at the density of 2 × 10^5^ cells/well and incubated for 16 h at 37 ◦C. The mAbs, used at a high concentration (150 nM), were added in the complete culture medium and incubated for 72 hours. After treatment, the supernatants were analyzed for measuring LDH release and cell viability was measured by the trypan blue exclusion test^39^. Cell survival was expressed as percent of viable cells in the presence of the mAbs with respect to the control cells untreated or treated with an unrelated IgG4, used as a negative control.
